# Expression of concern to ‘Depletion of TDP 43 overrides the need for exonic and intronic splicing enhancers in the human apoA-II gene’

**DOI:** 10.1093/nar/gkad113

**Published:** 2023-02-11

This is an expression of concern about: *Nucleic Acids Research*,Volume 33, Issue 18, 1 October 2005, Pages 6000–6010, https://doi.org/10.1093/nar/gki897

The Editors were alerted in July 2022 about potential issues with Figures 2C and 3B as detailed below.

- Figure 2C: ISE3wt SRp75 and ISE3wt SRp55 appear to be identical. ISE3m SRp75 and + SRp75 appear to be identical.
- Figure 3B: Lanes 5 and 6 appear to be identical.

The Editors have contacted the authors and are referring the matter to the institution where the research was conducted for investigation. In the interim, we advise readers to examine the details of this study with particular care.

Julian E. Sale, Barry L. Stoddard

Senior Executive Editors

